# Glycolysis reprogramming in cancer-associated fibroblasts promotes the growth of oral cancer through the lncRNA H19/miR-675-5p/PFKFB3 signaling pathway

**DOI:** 10.1038/s41368-021-00115-7

**Published:** 2021-03-25

**Authors:** Jin Yang, Xueke Shi, Miao Yang, Jingjing Luo, Qinghong Gao, Xiangjian Wang, Yang Wu, Yuan Tian, Fanglong Wu, Hongmei Zhou

**Affiliations:** 1 State Key Laboratory of Oral Diseases & National Clinical Research Center for Oral Diseases & National Center of Stomatology, West China Hospital of Stomatology, Sichuan University, Chengdu, China; 2 Department of Oral and Maxillofacial Surgery, West China Hospital of Stomatology, Sichuan University, Chengdu, China; 3 Department of Oral Medicine, the Second Affiliated Hospital, School of Medicine, Zhejiang University, Hangzhou, China; 4 Department of General Dentistry, the Second Affiliated Hospital, School of Medicine, Zhejiang University, Hangzhou, China

**Keywords:** Cancer microenvironment, Oral cancer, RNA

## Abstract

As an important component of the tumor microenvironment, cancer-associated fibroblasts (CAFs) secrete energy metabolites to supply energy for tumor progression. Abnormal regulation of long noncoding RNAs (lncRNAs) is thought to contribute to glucose metabolism, but the role of lncRNAs in glycolysis in oral CAFs has not been systematically examined. In the present study, by using RNA sequencing and bioinformatics analysis, we analyzed the lncRNA/mRNA profiles of normal fibroblasts (NFs) derived from normal tissues and CAFs derived from patients with oral squamous cell carcinoma (OSCC). LncRNA H19 was identified as a key lncRNA in oral CAFs and was synchronously upregulated in both oral cancer cell lines and CAFs. Using small interfering RNA (siRNA) strategies, we determined that lncRNA H19 knockdown affected proliferation, migration, and glycolysis in oral CAFs. We found that knockdown of lncRNA H19 by siRNA suppressed the MAPK signaling pathway, 6-phosphofructo-2-kinase/fructose-2,6-biphosphatase 3 (PFKFB3) and miR-675-5p. Furthermore, the lncRNA H19/miR-675-5p/PFKFB3 axis was involved in promoting the glycolysis pathway in oral CAFs, as demonstrated by a luciferase reporter system assay and treatment with a miRNA-specific inhibitor. Our study presents a new way to understand glucose metabolism in oral CAFs, theoretically providing a novel biomarker for OSCC molecular diagnosis and a new target for antitumor therapy.

## Introduction

Cancer-associated fibroblasts (CAFs), a dominant component in the tumor stroma, exhibit a promotive role in tumors, including breast, prostate, and oral cancers.^1,2^ By the curettage method combined with trypsinization, we previously separated human oral CAFs from oral tumors and identified their specific phenotypes and characteristics.^3^ It is becoming increasingly evident that cancer initiation, progression, and invasion are affected by “cross-talk” between CAFs and cancer cells rather than depending solely on autonomous cancer cell defects.^4,5^ Among the “cross-talk”, given studies have shown that metabolic changes in CAFs can facilitate metabolic reprogramming and tumor progression,^6–8^ and caveolin-1 (CAV-1)- and monocarboxylate transporter-4 (MCT-4)-induced glycometabolic reprogramming in fibroblasts was found to be activated by tumoral microvesicles to promote tumor progression.^9^ However, Jensen et al. held that CAV-1- and MCT-4-induced reverse Warburg metabolism in oral cancer was not dependent upon myofibroblasts.^10^ This evidence suggests that CAF-mediated glycometabolism and its role in supplying energy to cancer cells remain controversial.

Long noncoding RNAs (lncRNAs) are generally defined as a class of transcripts longer than 200 nucleotides that lack protein-coding potential^11^ but have a critical effect on regulatory processes at the post-transcriptional and pre-transcriptional levels. Accumulating studies have supported the notion that abnormal regulation of lncRNAs contributes to tumor progression, metastasis, and glucose metabolism.^12^ Several lncRNAs, including lncRNA NBR2,^13^ lncRNA PVT1,^14^ and lncRNA H19,^15^ have been found to promote glycolysis in cancer. Mechanistically, these metabolic lncRNAs can directly regulate the expression of enzymes, regulatory molecules, and oncogenes and/or indirectly modulate the potential signaling pathways involved in glycometabolic processes, such as glucose uptake and transport and the tricarboxylic acid cycle (TCA cycle).^16^ Through knockdown of Lnc-CAFs in oral cancer, Ding et al. provided data demonstrating that Lnc-CAFs can transfer normal fibroblasts (NFs) to CAFs and maintain the CAF phenotype, subsequently promoting the tumor growth of oral cancer.^17^ However, to the best of our knowledge, few studies have been conducted to explore whether oral CAFs modulate tumor biological characteristics through lncRNAs.

To date, through deeper and more sensitive RNA sequencing, improved epigenomic technologies, and computational prediction techniques, the total number of identified lncRNAs has reached tens of thousands.^18^ The most controversial aspect of the study design is the difficulty of locating the “Achilles’ heel” targeting the metabolic biological characteristics of oral CAFs among these thousands of lncRNAs. In 2014, we developed a systems biology strategy that progressively cycles experiments and computations to discover epithelial–mesenchymal common targets (EMCTs), the common “fragile key points” for both the cancerous epithelium and its adjacent stroma.^19^ However, due to the differences between lncRNAs and proteins,^20–22^ we must improve the technology strategy to identify the key lncRNAs in oral CAFs and further predict their potential biological function in glucose metabolism. In this study, by performing in vitro and in vivo experiments, we targeted lncRNA H19 as a potential EMCT and highlight its potential value in the development of a therapeutic approach for oral cancer.

## Results

### Comparison of lncRNA and mRNA expression profiles in oral CAFs

**Fig. 1:**
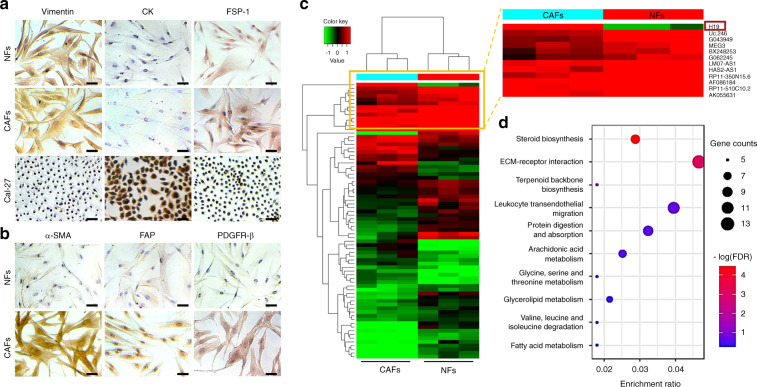
Primary culture, immunocytochemistry identification, and RNA sequencing of NFs and CAFs in OSCC. **a** The phenotypes of NFs, CAFs, and Cal-27 cells were determined by staining for specific markers of stromal mesenchymal cells (vimentin, FSP-1) and epithelial cells (cytokeratin, CK) via immunocytochemistry. Scale bars: 50 μm. Representative images from each group are presented. **b** The phenotypes of NFs and CAFs were determined by staining for specific markers of CAFs (α-SMA, FAP, and FDGFR-β) via immunocytochemistry. Scale bars: 50 μm. **c** Heatmap of the differentially expressed lncRNAs between CAFs and NFs. *n* = 3, *P* < 0.05, fold change >2. **d** Bubble chart showing the enriched KEGG pathway analysis terms of differentially expressed genes between CAFs and NFs

To determine the differentially expressed lncRNA profiles between NFs and CAFs, in this study, we enrolled 12 patients, 6 with oral cancer (Supplementary Table 1) and 6 who received a third molar extraction. Through primary cell culture and immunocytochemistry (ICC) staining, NFs and CAFs were found to be positive for vimentin and fibroblast specific protein-1 (FSP-1). NFs and CAFs were negative for cytokeratin (CK), while Cal-27 cells exhibited positive staining (Fig. 1a). Further examination of CAFs and NFs via ICC and immunofluorescence staining revealed that the expression levels of α-smooth muscle actin (α-SMA), fibroblast activated protein (FAP) and β-platelet-derived growth factor receptor (PDGFR-β) were higher in CAFs than in NFs (Figs. 1b and S1).

**Fig. 2:**
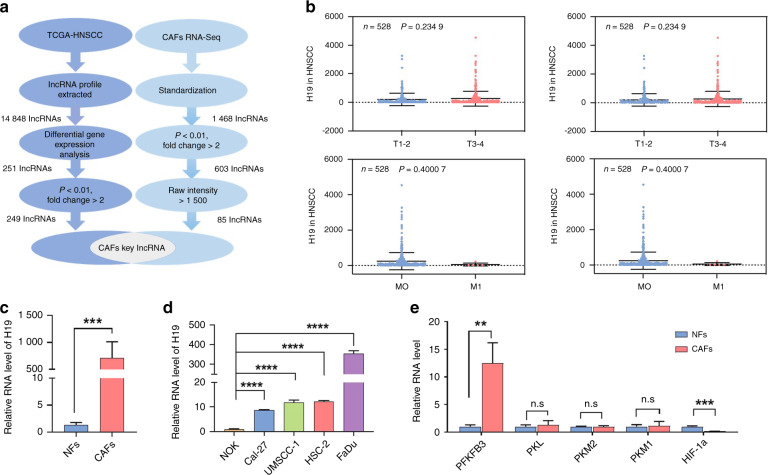
lncRNA H19 might be a key lncRNA seed in oral CAFs and is consistently upregulated in the epithelium and stroma. **a** The strategy for oral CAFs key lncRNA discovery. The two-flow procedure implemented in this study is shown. The left flow identifies lncRNA candidates serving in the epithelium, and the right flow searches for potential targets in the stroma. Two flows were crossed to obtain the key lncRNAs in oral CAFs, which might be identified as seed predictions of EMCTs. **b** Distribution of lncRNA H19 levels for different TNM statuses in HNSCC cases. Box plot showing that there was no significant association between TNM status and lncRNA H19 level (*n* = 528). **c** RT-PCR was performed to estimate the relative lncRNA H19 levels between NFs and CAFs (*n* = 6). **d** RT-PCR was performed to estimate the relative lncRNA H19 levels between Cal-27, UMSCC-1, HSC-2, FaDu, and NOK cells (*n* = 3). **e** RT-PCR was performed to estimate the relative RNA levels of enzymes involved in glucose metabolism, including PFKFB3, PKL, PKM2, PKM1, and HIF-1α, between NFs and CAFs (*n* = 3). Error bars, mean ± standard deviation (SD); n.s., not significant, **P < 0.01, ***P < 0.001, ****P < 0.000 1; by *t*-test

To explore whether lncRNAs and mRNAs are expressed differently between NFs and CAFs, we performed RNA sequencing in NFs and CAFs (both at passage 3) derived from patients with oral cancer without lymph node metastasis who underwent no preoperative chemotherapy and/or radiotherapy. As shown in Fig. 1c and Fig. S1b, our results suggested that the lncRNA/mRNA profiles were significantly different between NFs and CAFs. The differentially expressed mRNAs with a fold change above 2 and a false discovery rate (FDR) less than 0.05 were selected for further analysis. Since different mRNAs were predicted to have different biological functions, including glucose metabolism, via Kyoto Encyclopedia of Genes and Genomes (KEGG) pathway enrichment analysis, we found that one of the major phenotypic differences between oral CAFs and NFs (upregulated differentially expressed genes (DEGs) in oral CAFs) was reflected in the metabolism and synthesis of substances (Fig. 1d). To study these microarray data, more stringent filtering criteria (fold change >2, *P* < 0.01, raw signal intensity >1500)^23^ were used in the study. In total, we found that 85 lncRNAs were upregulated significantly in CAFs compared to NFs (Fig. 2a).

### Prediction and validation of H19 as a key lncRNA seed in oral CAFs

To explore the EMCTs of lncRNAs, we downloaded the gene expression profiles and clinical information of all 528 patients with head and neck squamous cell carcinoma (HNSCC) from the TCGA database. Among them, 142 (27.5%) patients were female, and 386 (73.1%) patients were male. 14 848 lncRNAs were extracted and by comparing Affymetrix microarray data of the HNSCC samples in TCGA database to reveal differences in gene expression profiles between cancer and normal tissues, we found 251 DEGs in lncRNA profile. Then, we screened out 249 lncRNAs with fold change >2 and *P* < 0.01 (Fig. 2a). Combined with our data from the tumor stroma, lncRNA H19 was identified as a seed prediction of EMCTs and a key lncRNA in oral CAFs (Fig. 2a). Additionally, we plotted the distribution of lncRNA H19 levels based on the TNM status and stage in HNSCC cases. Unlike what we expected, the average lncRNA H19 levels in HNSCC exhibited no significant difference among tumor volumes, stages, lymph node metastasis, and distant metastasis (Fig. 2b).

To confirm microarray data, 6 pairs of patients with or without oral cancer were included for isolation of NFs and CAFs. Real-time PCR (RT-PCR) data revealed that lncRNA H19 was augmented significantly in oral CAFs (Fig. 2c). Intriguingly, through RT-PCR, we found that lncRNA H19 in several oral cancer cell lines, including Cal-27, UMSCC-1, HSC-2 and FaDu cells, was significantly increased compared to the level in normal oral keratinocytes (NOKs) (Fig. 2d). Then, to address whether enzymes involved in glucose metabolism were different between oral CAFs and NFs, we included 6-phosphofructo-2-kinase/fructose-2,6-biphosphatase 3 (PFKFB3), pyruvate kinase L (PKL), pyruvate kinase M1/2 (PKM1/PKM2) and hypoxia inducible factor-1α (HIF-1α) for RT-PCR analysis. Our results indicated that PFKFB3 expression was increased and HIF-1α expression was attenuated in oral CAFs (Fig. 2e).

### LncRNA H19 knockdown affected glycolysis, proliferation, and migration in oral CAFs

**Fig. 3:**
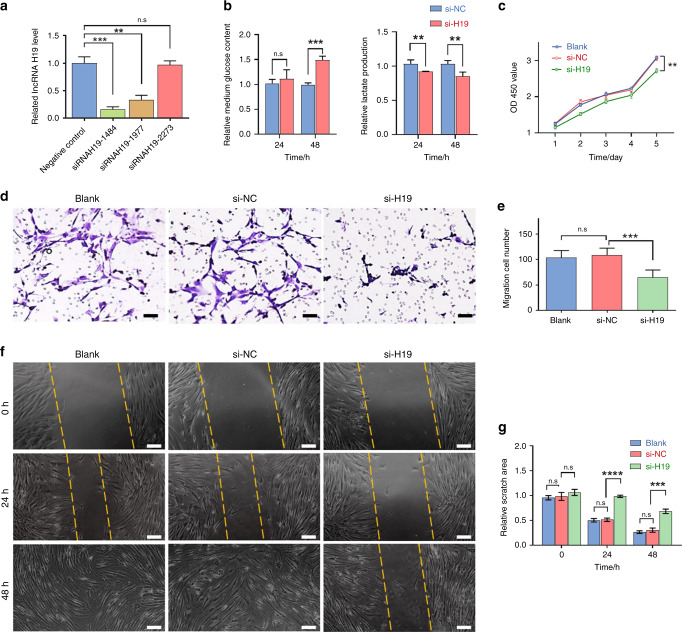
lncRNA H19 knockdown suppressed oral CAF glycolysis, proliferation, and migration in vitro. **a** RT-PCR was performed to estimate the relative lncRNA H19 levels between CAFs infected with si-H19 (siRNAH19-1484, siRNAH19-1977, and siRNAH19-2273) and the NC (*n* = 3). **b** Compared to NC cells, lncRNA H19 downregulation attenuated glucose uptake and LD production in oral CAFs based on glucose assays, and LD assays, with a significant difference at 48 h (*n* = 3). **c** CCK-8 assays performed at the indicated times (*n* = 3). **d** Transwell assays were performed to evaluate cell migration. Scale bars: 100 μm. **e** The statistical graph indicates the average number of cells in 9 random high-power fields. The results are presented as the mean ± SD from three independent experiments (*n* = 3). **f** Representative images of wound-healing assays at the indicated times after scratching. Scale bars: 200 μm. **g** The relative breadth of the wound after migration was measured and calculated among the groups (*n* = 3). Error bars indicate the mean ± SD; n.s., not significant, **P < 0.01, ***P < 0.001, ****P < 0.000 1; by *t*-test or two-way ANOVA

To investigate the effects of lncRNA H19 in oral CAFs, we used siRNAH19-1484, named si-H19, to knockdown lncRNA H19 expression (Fig. 3a). Link-Finder^21^ was used to explore the relationship between lncRNA H19 and mRNA expression in TCGA HNSCC cohort (*n* = 528). We performed KEGG pathway enrichment analysis and found that 2 678 genes were closely correlated with lncRNA H19 (FDR < 0.01). As shown in the volcano plot (Fig. S2a), 1 669 genes (dark red dots) had a significant positive correlation with lncRNA H19, whereas 1 009 genes (dark green dots) displayed a significant negative correlation (FDR < 0.01, *t-*test followed by multiple testing correction). As shown in Fig. S2b, the glycolysis/gluconeogenesis pathway was enriched among these genes, predicting a potential role of lncRNA H19 in regulating glycolysis/gluconeogenesis.

We next analyzed the function of lncRNA H19 in regulating glycolysis in oral CAFs. Using glucose and lactate assays, we found evidence that lncRNA H19 knockdown decreased glucose uptake and lactate secretion (Fig. 3b and Fig. S2c). To further address the functional impact of lncRNA H19 in oral cancer, we used a CCK-8 array and found that lncRNA H19 knockdown inhibited the proliferation of oral CAFs (Fig. 3c). Migration analysis using a Transwell assay revealed that downregulation of lncRNA H19 suppressed oral CAF migration (si-H19 vs. si-NC, *P* = 0.000 4, Fig. 3d, e). Additionally, wound-healing assays demonstrated that lncRNA H19 knockdown attenuated the migration ability of oral CAFs compared with the controls (si-H19 vs. si-NC, 24 h: *P* < 0.000 1, 48 h: *P* = 0.000 7, Fig. 3f, g).

### MiR-675-5p/PFKFB3 is involved in lncRNA H19-mediated glycolysis in oral CAFs

**Fig. 4:**
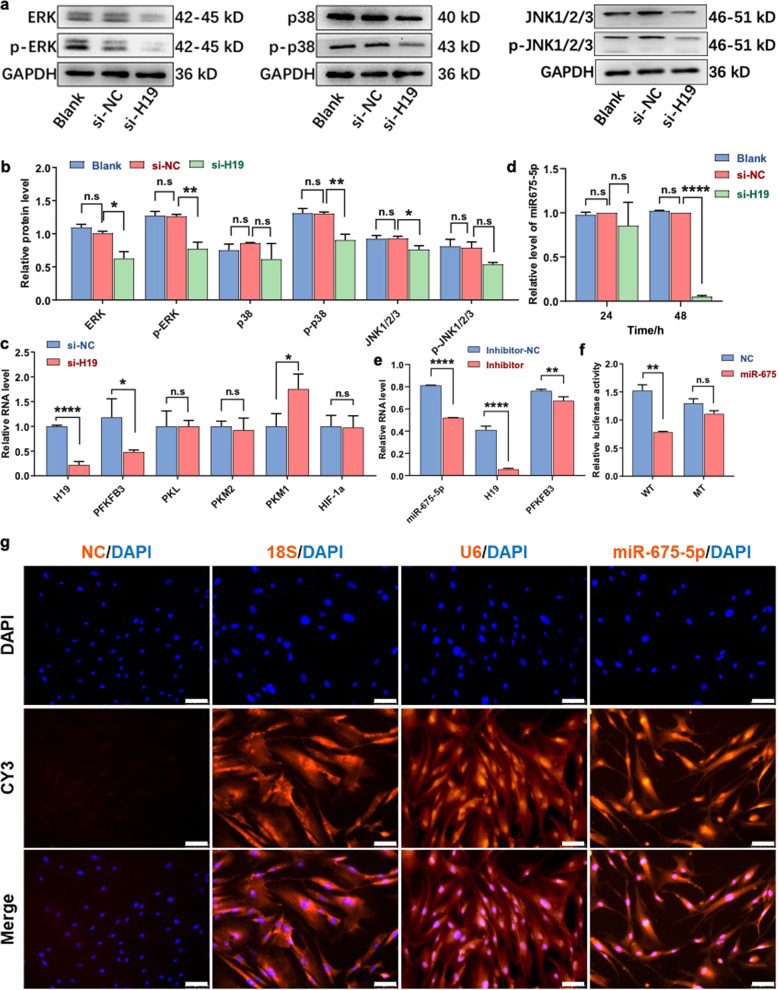
miR-675-5p/PFKFB3 act as key regulators in lncRNA H19-mediated glycolysis in oral CAFs. **a**, **b** At the protein level, inhibition of lncRNA H19 significantly decreased ERK, p-ERK, p-p38, and JNK1/2/3 levels in the si-H19 group (*n* = 3). **c** RT-PCR was performed to estimate the relative RNA levels of lncRNA H19 and enzymes involved in glucose metabolism, including PFKFB3, PKL, PKM2, PKM1, and HIF-1α, compared to those in NC cells; a significant difference found was found in the levels of lncRNA H19 and PFKFB3 (*n* = 3). **d** Compared to NC cells and blank controls, the relative RNA levels of miR-675-5p were decreased in the si-H19 group based on RT-PCR results (*n* = 3). **e** After miR-675-5p knockdown, RT-PCR showed that the relative RNA levels of lncRNA H19 and PFKFB3 were decreased in the inhibitor group (*n* = 3). **f** Luciferase reporter assay showing that miR-675-5p targets the PFKFB3 gene directly (*n* = 3). **g** Compared to 18S (cytoplasmic), U6 (nuclear) and NC (nonspecific) controls, RNA-FISH confirmed the nuclear localization of miR-675-5p. Scale bars: 75 μm. Error bars, mean ± SD; n.s., not significant, *P < 0.5, **P < 0.01, ***P < 0.001; by *t*-test

Since MAPK signal transduction cascades are one of the most classical glycolysis pathways,^24^ we examined the relationships between lncRNA H19 and the ERK-MAPK, p38-MAPK, and JNK MAPK signaling pathways. Additionally, TCGA data showed that the gene levels of ERK-MAPK, p38-MAPK, and JNK-MAPK tended to rise with an increase in H19 expression (Fig. S3a). Through western blotting, we found that lncRNA H19 knockdown significantly reduced the expression of ERK, p-ERK, p-p38, and JNK compared with that in the NC group (Fig. 4a, b).

To explore whether lncRNA H19 interacted with glucose metabolic enzymes, we detected the relative mRNA levels of key glycolytic enzymes compared with levels in the control. At the mRNA level, PFKFB3 was downregulated and PKM1 was upregulated in oral CAFs with lncRNA H19 knockdown compared to controls (Fig. 4c). LncRNA H19 also exerts its diverse actions by interacting with microRNAs (miRNAs) as a sponge or as a precursor.^25^ Furthermore, we detected the miRNAs associated with lncRNA H19, as shown in the heat maps (Fig. S3b) and scatter plots (Fig. S3c). Hsa-miR-675 was closely correlated with lncRNA H19 (Spearman’s correlation = 0.923 9). The mature miRNAs miR-675-3p and miR-675-5p, derivatives of lncRNA H19,^26^ have been found to be involved in cancer development.^25^ At the RNA level, we found that lncRNA H19 knockdown significantly reduced the expression of miR-675-3p and miR-675-5p (Figs. S3d and 4d). After knockdown of miR-675-5p, we found that lncRNA H19 and PFKFB3 showed a significant reduction compared with controls (Fig. 4e). Then, a luciferase reporter system showed that miR-675-5p targeted the PFKFB3 gene directly (Fig. 4f). Based on these data, lncRNA H19 might increase the transcription level of the PFKFB3 gene through miR-675-5p in oral CAFs.

To further address the role of miR-675-5p in the PFKFB3 gene-mediated glycolysis pathway, SEdb,^27^ TargetScan (www.targetscan.org) and RPmirDIP^28^ were used to study the association between super-enhancer regions of PFKFB3 and the predicted consequential hsa-miR-675-5p target region (Fig. S3e). A localization study in oral CAFs using RNA-FISH further confirmed nuclear localization of miR-675-5p compared with the cytoplasmic localization of 18S and the nuclear localization of U6 (Fig. 4g). Importantly, glucose and lactate assays of culture medium (CM) showed that knockdown of miR-675-5p decreased glucose uptake and lactate secretion (Fig. S3f). In addition, miR-675-5p knockdown decreased the enzymatic activity of intracellular lactate dehydrogenase (Fig. S3f). Our results indicate that lncRNA H19-derived miR-675-5p acts on the PFKFB3 gene-mediated glycolysis pathway in oral CAFs.

### LncRNA H19 knockdown in CAFs suppressed tumor growth in vivo

**Fig. 5:**
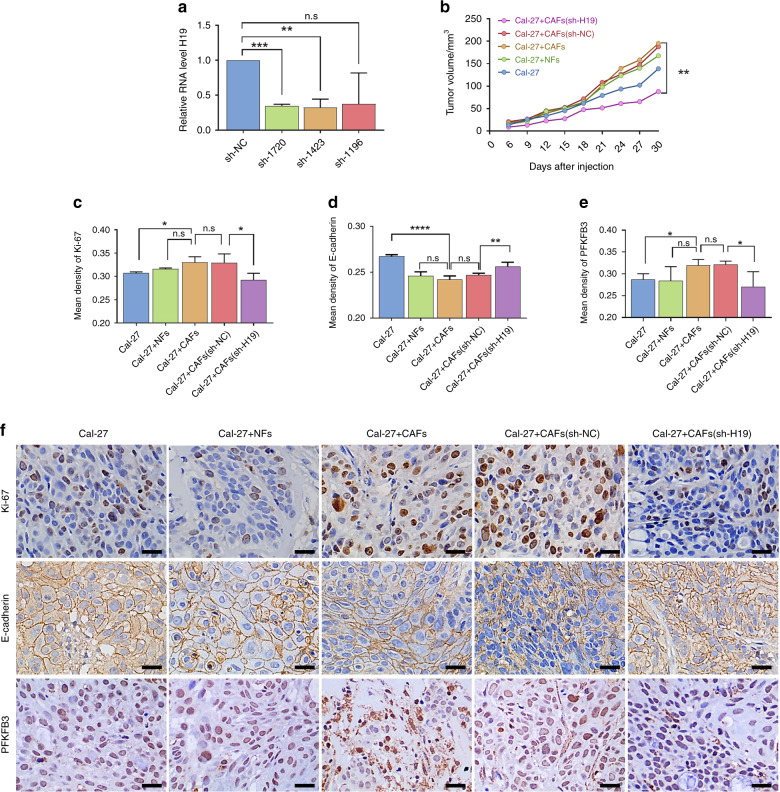
Knockdown of lncRNA H19 suppressed tumor growth in vivo. **a** RT-PCR was performed to estimate the relative lncRNA H19 levels between CAFs infected with sh-H19 (sh-1720, sh-1423, and sh-1196) and the negative control (sh-NC). In addition, the sh-1720 cells were renamed sh-H19 (*n* = 3). **b** Tumors were measured with calipers, and volumes were calculated every week; the line chart shows the differences (*n* = 4). **c**–**e** The mean density (MD) of Ki-67, E-cadherin, LDH and PFKFB3 is expressed as the mean ± SD. **f** Representative immunohistochemistry staining of Ki-67, E-cadherin, and PFKFB3 in tumors from the nude mouse transplanted model. Scale bars: 20 μm. *n* = 4; error bars, mean ± SD; n.s., not significant, *P < 0.5, **P < 0.01, ***P < 0.001, ****P < 0.000 1; by *t*-test

To further examine the effect of lncRNA H19 on tumor progression and the PFKFB3 gene-mediated glycolysis pathway in vivo, we established a nude mouse transplanted tumor model and used shRNAH19-1720 (named sh-H19) to knockdown the expression of lncRNA H19 in oral CAFs (Fig. 5a). The tumor volumes in the Cal-27 + CAFs group were larger than those in the controls, and lncRNA H19 knockdown exhibited a suppressive role in tumor growth (Fig. 5b and S4a). Through immunohistochemistry (IHC), we found that Ki-67 was augmented while E-cadherin was reduced (Figs. 5c, d) in Cal-27 + CAF-bearing tumors compared to Cal-27-bearing tumors (Fig. 5f). Furthermore, lncRNA H19 knockdown reduced Ki-67 expression (Fig. 5c) and increased E-cadherin expression (Fig. 5d).

**Fig. 6:**
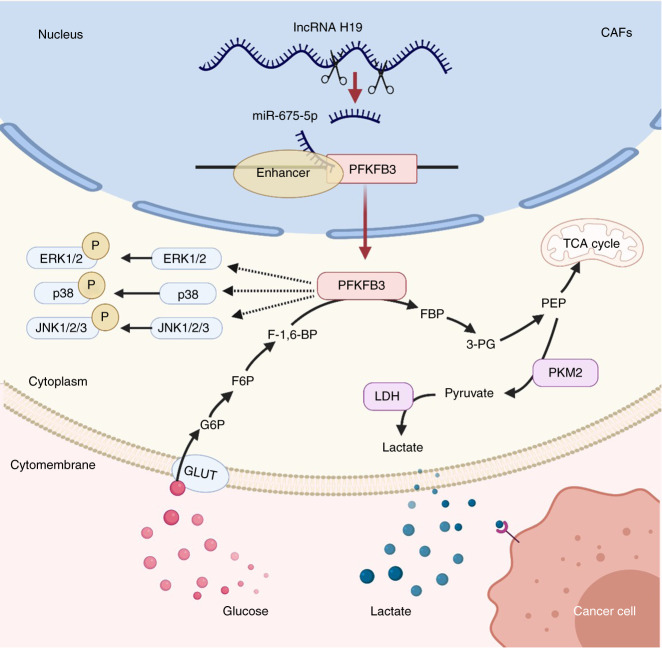
Schematic overview of lncRNA H19 how mediates glycolysis via miR-675-5p/PFKFB3 in oral CAFs. GLUT, glucose transporters; G6P, glucose-6-phosphate; F6P, fructose 6-phosphate; F-1,6-BP fructose 1,6 bisphosphates, FBP fructose 1,6-bisphosphatase; 3-PG, 3-phosphoglycerate; PEP, phosphoenolpyruvate. Created with BioRender.com

Moreover, we validated the glycolytic pathway in transplanted tumors. The levels of lactate dehydrogenase (LDH) exhibited no significant difference between the Cal-27 + CAF group and the Cal-27 group (Fig. S4c). Downregulation of PFKFB3 was detected in the Cal-27 + CAF group compared with the Cal-27 group (Fig. 5e). After lncRNA H19 knockdown, lower LDH and PFKFB3 expression was detected in the CAF (sh-H19) group than in the control group (Figs. 5e, f and S4b). In summary, these results indicate that the lncRNA H19/PFKFB3 pathway might be involved in oral CAF-mediated glycolysis in vivo (Fig. 6).

## Discussion

LncRNAs mediate glucose metabolism reprogramming in cancer mainly by regulating metabolic enzymes directly and/or by mutual regulation with oncogenes or tumor suppressors indirectly.^12^ As an emerging hallmark of cancer, metabolic interactions are an important aspect of crosstalk in the tumor stroma,^29^ and CAF metabolic reprogramming can promote tumor progression.^6–8^ In this study, we found that lncRNA H19, the first discovered riboregulatory lncRNA, is a potential EMCT candidate with antitumor activity. We demonstrated that lncRNA H19 knockdown in oral CAFs not only attenuated glycolysis and affected cellular biological behaviors but also suppressed tumor progression in oral cancer.

Ample evidence has supported the notion that metabolic reprogramming provides tumors with the ability to survive and proliferate.^30,31^ In OSCC, Cai et al. reported that LDH facilitates glycolysis and epithelial–mesenchymal transition in cancer cells.^32^ Several inhibitors of glycolysis enzymes were effective in suppressing the proliferation of cancer cells and have been in clinical trials,^33^ indicating that targeted epithelial glycolysis exhibits great potential in the clinic. However, the given studies have focused predominantly on the altered metabolism of cancer cells, but the reciprocal regulation of metabolic alterations between cancer cells and CAFs is thought to be a critical mechanism underlying the regulation of cancer progression. As the “soil” of tumors, CAFs conduct aerobic glycolysis similar to cancer cells and “feed” cancer cells directly.^6^ Glycolysis in breast CAFs was found to be dependent on the expression of the metabolic genes ENO2, HK2, and PFKFB3.^34^ Additionally, in lung cancer, CAFs were reported to reprogram metabolic pathways involving ROS and TGF-β signaling.^7^ Similarly, normal oral fibroblasts acquired a glycolysis metabolic phenotype through microvesicle or mitochondrial exchange when cocultured with OSSC cells.^9,35^ In this study, we provide evidence that the PFKFB3 mRNA level is higher in oral CAFs than in NFs (Fig. 2e). Thus, all these data suggest that CAF-mediated glycolysis is a common event in tumors, including oral cancer.

To date, many studies have explored glucose metabolism reprogramming in tumor cells and CAFs at the post-transcriptional level^7,9,32,34–37^; however, at the pre-transcriptional level, the roles of lncRNAs in CAF-mediated glycolysis remain largely unknown. The number of lncRNA genes far exceeds the number of protein-coding genes,^38^ and lncRNAs exhibit unique expression profiles in specific cell types or specific cancer types,^39^ indicating that lncRNAs might play a critical role in cancer glucose metabolism reprogramming. In support of this notion, Wang et al. reported that lnc-p23154 was associated with the metastatic phenotype of OSCC cells and participated in glycolysis.^40^ Furthermore, downregulation of miR-26b in breast cancer CAFs promoted cell migration and invasion through the downstream Rho GTPase-mediated glycolytic pathway,^41^ suggesting that lncRNAs located upstream of miRNAs might also affect glycolysis in CAFs. In the present study, we found that lncRNA H19 knockdown suppressed the glycolysis state of oral CAFs.

It is generally accepted that cells that show active proliferative capacity prefer glycolysis even when oxygen is present to meet fast growth and proliferation demands, which is known as the “Warburg effect”,^42,43^ but the roles of lncRNA H19 in affecting the biological hallmarks of CAFs have not been well addressed.^44^ To date, lncRNA H19 has been reported to sustain cell proliferation in cancer. Mechanistically, lncRNA H19 facilitates the G1/S transition to promote cell proliferation in esophageal squamous cell carcinoma,^45^ and miR-675-5p induced by lncRNA H19 suppressed p53 protein, leading to increased cell proliferation in non-small cell lung cancer.^46^ Similarly, in this study, using a CCK-8 assay, we found that lncRNA H19 promoted oral CAF proliferative phenotypes. Interestingly, lncRNA H19 knockdown in oral CAFs suppressed tumor growth in vivo, indicating that lncRNA H19 expressed by oral CAFs was associated with the ability of cancer cells to sustain proliferation.

In addition, lncRNA H19 can increase cell migration in cancer.^25^ Yang et al. indicated that lncRNA H19 regulated the migration of colon cancer cells through the miR138/high-mobility group A protein pathway.^47^ Zhang et al. demonstrated that lncRNA H19 can induce migration and invasion in colorectal cancer cells by directly binding to hnRNPA2B1.^48^ Similar to studies related to epithelial cells, we found that lncRNA H19 promoted the migration of oral CAFs in vitro. Furthermore, our in vivo tumor metastasis study results revealed that lncRNA H19 knockdown in oral CAFs increased E-cadherin in transplanted tumors. CAF-guided collective cell migration contributes to cancer metastasis, and E-cadherin is required for force transmission between CAFs and cancer cells,^49,50^ indicating that cancer cells invading the surroundings follow the migration of CAFs. Induction of epithelial-to-mesenchymal transition (EMT) is also an important mechanism in the prometastatic effect of CAFs.^51^ The CM of CAFs can repress expression of epithelial markers, such as E-cadherin, thereby inducing EMT in bladder cancer.^52^ The downregulation of E-cadherin in transplanted tumors might be attributed to EMT induced by oral CAFs and promote metastasis; however, the role of lncRNA H19 in CAFs in oral cancer metastasis still needs to be further studied.

However, unlike what we expected, the absence of a relationship between lncRNA H19 expression and poor clinical features of HNSCCs could be a barrier to identifying EMCTs. Actually, HNSCCs are unexpectedly heterogeneous in nature^53^ and arise from the squamous epithelium of the oral cavity, oropharynx, larynx, and hypopharynx.^54^ Hong et al. reported that lncRNA H19 was upregulated in OSCC tissues and associated with clinical features, such as TNM stage, nodal invasion, and shorter overall survival of patients.^55^ Interestingly, Vishwakarma et al. showed downregulated expression of lncRNA H19 in tumor tissues of OSCC patients.^56^ Contrary findings concerning the respective levels of H19 expression in OSCC may be due to differences in the number and composition of the tissues used, while infiltrating stromal and immune cells are also the key components of tumor tissue.^57^ Contrary findings on lncRNA H19 mechanisms of action have also been reported in pancreatic cancer.^25,58^ We found elevated lncRNA H19 expression levels in human OSCC cell lines, indicating that lncRNA H19 is a seed EMCT.

Further, we must determine the potential pathways related to the lncRNA H19 involvement in glycolysis in oral CAFs. Curtis et al. showed that metastasizing ovarian cancer cells can mobilize glycogen following their interaction with CAFs, which was dependent on p38-MAPK activation in CAFs, leading to increased proliferation, invasion, and metastasis.^59^ We also found positive correlations between lncRNA H19 and the gene levels of ERK-MAPK, p38-MAPK, and JNK-MAPK. Since the MAPK signaling cascades can be regulated by lncRNA H19, the proteins targeted by lncRNA H19 might be located in upstream pathways. Importantly, a correlation between PFKFB3 and lncRNA H19 was found in this study. PFKFB3 has high kinase activity to shunt glucose toward glycolysis.^60^ As a vital regulator of glycolysis, accumulating studies have suggested that PFKFB3 is associated with many aspects of cancer.^61^ Pharmacological inhibition of PFKFB3 can suppress tumor growth and alleviate metastasis in HNSCC.^62^ PFKFB3-dependent infiltration of lymphotoxin-alpha in HNSCC promoted the proliferation and migration of human umbilical vein endothelial cells, which may contribute to aberrant angiogenesis.^63^ The PFKFB3-mediated glycolysis pathway plays an important role in the influence of lncRNA H19 on glucose metabolism reprogramming in oral CAFs.

Although LINC00092 can bind PFKFB3 directly,^64^ the binding site between lncRNA H19 and PFKFB3 is still unknown. LncRNAs participate in cancer progression as miRNA decoys or targets. For instance, He et al. reported that lncRNA UCA1/miR-182/PFKFB2 modulated glioblastoma-associated stromal cell-mediated glycolysis.^65^ In this study, using TargetScan (www.targetscan.org) and a luciferase reporter system, we provide evidence that PFKFB3 is a target gene of lncRNA H19-derived miR-675-5p. Unlike the ceRNA hypothesis,^66^ we found that the RNA levels of PFKFB3 and miR-675-5p were downregulated together in oral CAFs when lncRNA H19 was knocked down. However, miRNAs localized in the nucleus can positively activate gene expression.^67,68^ Some nuclear-localized miRNAs can promote the activation of oncogenes by binding promoters or enhancers.^69^ After analysis of the association between super-enhancer regions of PFKFB3 and the miR-675-5p target region and confirmation of the nuclear localization of miR-675-5p, the mechanism of lncRNA H19-derived miR-675-5p acting on PFKFB3 might be verified by the NamiRNA-enhancer-gene activation network. Seviour et al. revealed an activation function of miR-124 through binding to and inducing transcription on the p27 promoter region in breast and ovarian cancer cell lines.^70^ Costa et al. observed that miR-675-5p overexpression in normoxia promoted upregulation of some genes.^71^ Therefore, lncRNA H19-derived miR-675-5p might affect the PFKFB3-mediated glycolysis pathway and reprogram the glycolysis state in oral CAFs.

In conclusion, we have shown that there is a close relationship between lncRNA H19 and glycolysis in oral CAFs. Knockdown of lncRNA H19 suppressed glucose metabolism and cell proliferation and migration in oral CAFs. LncRNA H19-derived miR-675-5p might bind PFKFB3 to facilitate glycolysis in oral CAFs. Our results highlight the effect of lncRNAs on the biological hallmarks of oral CAFs, providing novel insight into the biology of OSCC and raising the possibility of utilizing EMCTs for clinical interventions for OSCC.

## Materials and methods

### Clinical tissue sample collection and primary cell culture

All samples were obtained from the West China Hospital of Stomatology at Sichuan University during 2017–2019. In total, 6 OSCC patients and 6 patients who received third molar extraction as normal controls were enrolled in our study. OSCC diagnoses were confirmed by hematoxylin and eosin staining by two experienced pathologists. The OSCC patients were 45–63 years old, experienced no relapses, and underwent no preoperative chemotherapy and/or radiotherapy (Supplementary Table 1). This study was approved by the Human Research Ethics Committee of West China Hospital of Stomatology at Sichuan University (No. WCHSIRB-D-2017-016).

Oral CAFs and NFs were isolated and cultured using the curettage method with trypsinization.^3^ Primary CAF cells were generated from the tumor tissues of OSCC patients. Primary NF cells were generated from normal tissue in patients who received a third molar extraction. After being washed with antibiotics, the tissues were cut into pieces approximately 1 mm in diameter and incubated at 37 °C with 5% CO_2_. The culture medium (CM) was Dulbecco’s modified Eagle’s medium with 10% FBS (Moregate Biotech, Bulimba, Australia).

### Plasmid construction and cell transfection

Small interfering RNA (siRNA) and short hairpin RNA (shRNA) sequences were designed and purchased from GenePharma (Suzhou, China) to target lncRNA H19. Knockdown of lncRNA H19 in oral CAFs was performed using liposome-mediated in vitro RNA transfection (Endofectin^TM^-Max, GeneCopoeia, Rockville, USA) with siRNA (named si-H19) and a negative control (named si-NC). Double strands of shRNA were inserted into the LV3-pGLV-h1-GFP-puro vector (GenePharma, Suzhou, China) after annealing, and the final construct for in vivo experiments was named sh-H19; the negative control was named sh-NC.

The mirVana^TM^ miRNA inhibitor was purchased from Thermo Fisher (Shanghai, China) to target hsa-miR-675-5p. In vitro knockdown of miR-675-5p in oral CAFs was performed using liposome-mediated RNA transfection (Endofectin^TM^-Max, GeneCopoeia, Rockville, USA) with mirVana^TM^ miRNA inhibitor (named inhibitor) and a negative control (named inhibitor-NC).

### Cell lines and mice

The human OSCC cell lines Cal-27, UMSCC-1, HSC-2, and FaDu were purchased from ATCC or obtained from State Key Laboratory of Oral Diseases. Further authentication was conducted via short tandem repeat DNA profiling analysis. Four- to six-week-old BALB/c nude mice, half male and half female, were purchased from Charles River (Beijing, China). To investigate the role of lncRNA H19 in OSCC, CAFs infected with sh-H19 and Cal-27 cells were used in the following test groups: CAFs infected with sh-NC and Cal-27 cells, CAFs and Cal-27 cells, and NFs and Cal-27 cells; Cal-27 cells were used alone as the control group in this study. These conditional cells were subcutaneously co-injected into the rear flank of nude mice (five per group). All experimental protocols were approved by the Research Ethics Committee of West China Hospital of Stomatology, Sichuan University (No. WCHSIRB-D-2017-187). The animals were cared for following the guidelines for the use and care of laboratory animals.

### RNA isolation, RNA sequencing, and qRT-PCR

Total RNA was isolated from primary cells at the third passage using TRIzol reagent (Tianmo Biotech, Beijing, China) and quantified by measuring the optical density 260/280 and optical density 260/230. For RNA sequencing, RNA integrity was assessed via standard denaturing agarose gel electrophoresis. We performed RNA sequencing with the help of KangChen Biotech (Shanghai, China). RNA labeling and array hybridization were performed according to the manufacturer’s protocol. The raw data can be accessed via GSE164473. For mRNA and lncRNA expression, cDNA was synthesized from total RNA using a PrimeScript^TM^ RT reagent kit with a gDNA Eraser kit (Takara, Shiga, Japan). For miRNA expression, total RNA was reverse transcribed according to the manufacturer’s instructions using a Mir-X^TM^ miRNA First Strand Synthesis Kit (Takara, Shiga, Japan). cDNA was quantified on a LightCycler®480 System (Roche, Pleasanton, CA, USA) using SYBR®Premix Ex TaqTMII (Takara, Shiga, Japan). Detailed information on the PCR primer pairs used in this study is provided in Supplementary Table 2. Probes for miR-675-3p and miR-675-5p were generated using All-in-One^TM^ miRNA qPCR Primer Manual and Validation Report (GeneCopoeia, Guangzhou, China). Changes in the target mRNA and lncRNA content relative to GAPDH and changes in the target miRNA content relative to U6 were determined using the comparative CT (ΔΔ CT) method.

### Immunohistochemistry and immunofluorescence

Third or fourth passage primary cells were inoculated on cell slides, and immunohistochemistry or immunofluorescence assays were performed. Briefly, slides of CAFs, NFs or Cal-27 cells were covered with 4% formaldehyde for 30 min at room temperature and then washed with PBS three times. Then, immunohistochemistry or immunofluorescence assays were performed using a biotin-streptomycin assay kit according to the manufacturer’s protocol (Solarbio Life Science, Beijing, China). The cells were incubated with primary antibodies (dilution: 1:200 or 1:300) overnight at 4 °C. For immunofluorescence assays, fluorochrome-conjugated secondary antibodies (dilution: 1:300) were used. Detailed information on the antibodies used in this study is provided in Supplementary Table 3.

### Histology and immunohistochemistry

Samples were dissected from mice 6 weeks after transplantation and fixed in 10% neutral buffered formalin immediately for 24 h. Then, fixed samples were embedded in paraffin and sectioned at 4 μm thickness following a general histology protocol. Hematoxylin-eosin staining was used for histological examination. The following steps were performed as previously described.^19^ Primary antibodies were incubated with samples at 4 °C overnight, and then a DAB Detection kit (ZSGB-BIO, Beijing, China) was used for the DAB chromogen. Detailed information on the antibodies used in this study is provided in Supplementary Table 3. Hematoxylin was used for nuclear staining, and neutral gum was used to cover the slide. The integrated optical density (IOD) and area of the entire image collected were determined using Image-Pro Plus 6.0. The optical density of each sample is reported as the average of 3 images.

### Western blotting

Exponentially growing cells were used to produce whole-cell protein extracts in lysis buffer (KeyGEN BioTECH, Jiangsu, China). Equal amounts of total protein were subjected to 10% SDS polyacrylamide gel electrophoresis separation. After transfer to Immun-Blot polyvinylidene fluoride membranes (BioRad, Hercules, CA, USA), the membranes were blocked with 2% BSA solution in TBST for 1 h. Then, primary antibodies against proteins were added, and GAPDH was used as a control. The membrane was placed at 4 °C and left overnight. On the second day, the membranes were placed in TBST and shaken for 10 min three times. After incubation with secondary antibodies (ZSGB-Bio, Beijing, China) for 60 min at room temperature and a wash in TBST, the signals were detected using an Easy ECL western blot kit (TransGen Biotech, China). Proteins were visualized using Chemidox XRS (BioRad, Hercules, CA, USA). Detailed information on the antibodies used in this study is provided in Supplementary Table 3.

### RNA-FISH and luciferase reporter assays

The RNA-Fluorescence In Situ Hybridization (FISH) assay was performed according to the manufacturer’s protocol (GenePharma, Suzhou, China). U6 (5′-TTTGCGTGTCATCCTTGCG-3′), 18S (5′-CTGCCTTCCTTGGATGTGGTAGCCGTTTC-3′) and NC (5′-TGCTTTGCACGGTAACGCCTGTTTT-3′) were used as cytoplasmic positive controls, nuclear positive controls and negative controls, respectively. Finally, the glass slides were observed via fluorescence microscopy (Leica, Wetzlar, Germany).

For luciferase reporter assays, 0.2 µg of firefly luciferase reporter plasmid, 0.1 µg of PFKFB3 expression vector and equal amounts of miR-675-5p or scrambled negative control RNA were used to cotransfect cells cultured in 24-well plates. The PFKFB3 vector was used as a transfection control. At 24 h post-transfection, cells were assayed using luciferase assay kits (Promega).

### Bioinformatic analysis

KEGG pathway enrichment analysis was performed using the online website DAVID and WEB-based GEne SeT AnaLysis Toolkit.^72,73^ LinkedOmics was performed to identify the relationship between lncRNA H19 and mRNA/miRNA expression in TCGA HNSCC cohort.^21^ The Sangerbox online tool (http://sangerbox.com/) was used to assess differential gene expression and matrix extraction.

### Statistical analysis

GraphPad Prism 8 software was used for all statistical analyses. Data were analyzed using a *t*-test (if not indicated otherwise) and presented as the means ± standard deviation; if *P* > 0.05, the difference was not considered statistically significant (n.s.).

## Supplementary information

Supplementary Table

Figure S1

Figure S2

Figure S3

Figure S4
