# Supplementary material for: Glycolysis reprogramming in cancer-associated fibroblasts promotes the growth of oral cancer through the lncRNA H19/miR-675-5p/PFKFB3 signaling pathway
Source: Int J Oral Sci. 2021 Mar 25;13:12. doi: 10.1038/s41368-021-00115-7 (PMC7991655; doi:10.1038/s41368-021-00115-7)
Supplement: Supplementary file 1 — Supplementary Table [file 41368_2021_115_MOESM1_ESM.docx]

Supplementary Table 1. Clinical histopathological features of OSCC cases

| Factors | | Number |
| --- | --- | --- |
| Gender | Male | 4 |
|  | Female | 2 |
| T stage  (tumor volume) | T1 | 2 |
|  | T2 | 3 |
|  | T3 | 1 |
|  | T4 | 0 |
| Lymph node metastasis | Negative | 4 |
|  | Positive | 2 |
| Distant metastasis | Negative | 5 |
|  | Positive | 1 |
| Differentiation | Poor | 1 |
|  | Mild | 3 |
|  | Well | 2 |

6 cases. 6 of OSCC tissues for CAFs’ isolation and primary cell culture. 3 of OSCC tissues for CAFs’ transfection in vitro. 3 of OSCC tissues for CAFs’ RNA-Seq.

Supplementary Table 2. Primer sequence

| Gene |  | Sequence |
| --- | --- | --- |
| H19 | Forward | 5’-TCTGTTTCTTTACTTCCTCCACG-3’ |
|  | Reverse | 5’-GGGTAGCACCATTTCTTTCAT-3’ |
| PFKFB3 | Forward | 5’-GGTGTGCGACGACCCTAC-3’ |
|  | Reverse | 5’-GTACACGATGCGGCTCTG-3’ |
| PKM1 | Forward | 5’-GAGGCAGCCATGTTCCAC -3’ |
|  | Reverse | 5’-TGCCAGACTCCGTCAGAACT -3’ |
| PKM2 | Forward | 5′-CAGAGGCTGCCATCTACCAC-3′ |
|  | Reverse | 5′-CCAGACTTGGTGAGGACGAT-3′ |
| PKL | Forward | 5′-CTGGTGATTGTGGTGACAGG-3′ |
|  | Reverse | 5′-TGGGCTGGAGAACGTAGACT-3′ |
| HIF-1α | Forward | 5’–GCCGCTGGAGACACAATCATA–3’ |
|  | Reverse | 5’–GGTGAGGGGAGCATTACATCAT–3’ |
| GAPDH | Forward | 5’–GAACGGGAAGCTCACTGG–3’ |
|  | Reverse | 5’–GCCTGCTTCACCACCTTCT–3’ |

Supplementary Table 3 Antibody information

| Antibody | Company | Purpose |  |
| --- | --- | --- | --- |
| Anti-α-smooth muscle actin antibody | Abcam, UK | ICC | 1:200 |
| Anti-fibroblast activated protein antibody | Abcam, UK | ICC | 1:200 |
| Anti-Vimentin antibody | Abcam, UK | ICC | 1:200 |
| Anti-Cytokeratin antibody | Abcam, UK | ICC | 1:300 |
| Anti-Fibroblast Specific Protein-1 antibody | HUABIO, China | ICC | 1:200 |
| Anti-Platelet derived growth factor receptor-β antibody | HUABIO, China | ICC | 1:250 |
| Anti-Ki-67 antibody | Abcam, UK | IHC | 1:50 |
| Anti-E-cadherin antibody | Abcam, UK | IHC | 1:50 |
| Anti-PFKFB3 antibody | HUABIO, China | IHC | 1:50 |
| Anti-lactate dehydrogenase antibody | HUABIO, China | IHC | 1:50 |
| Anti-p38 antibody | HUABIO, China | WB | 1:1000 |
| Anti-Phospho-p38 antibody | HUABIO, China | WB | 1:1000 |
| Anti-ERK1/2 antibody | HUABIO, China | WB | 1:1000 |
| Anti-Phospho-Erk1+Erk2 antibody | HUABIO, China | WB | 1:1000 |
| Anti-JNK1+JNK2+JNK3 antibody | HUABIO, China | WB | 1:500 |
| Anti-Phospho-JNK1/2/3 antibody | HUABIO, China | WB | 1:1000 |
