# Supplementary figures and images for: Glycolysis reprogramming in cancer-associated fibroblasts promotes the growth of oral cancer through the lncRNA H19/miR-675-5p/PFKFB3 signaling pathway
Source: Int J Oral Sci. 2021 Mar 25;13:12. doi: 10.1038/s41368-021-00115-7 (PMC7991655; doi:10.1038/s41368-021-00115-7)

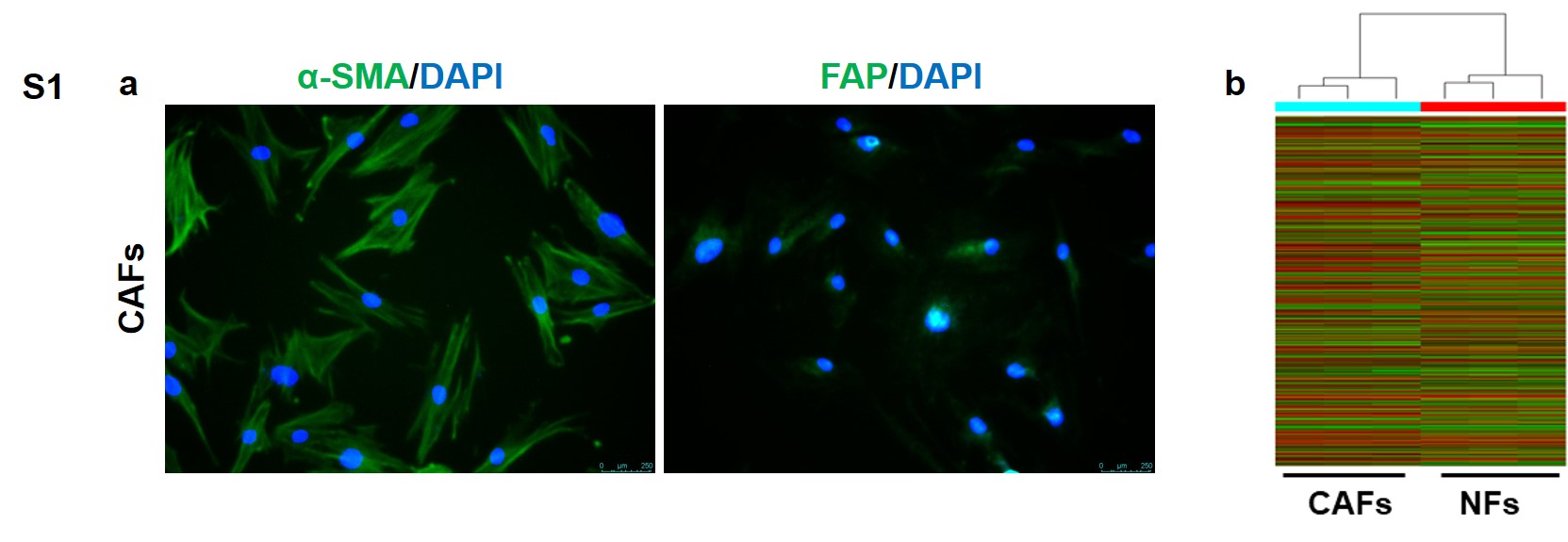

Supplement: Supplementary file 2 — Figure S1 [file 41368_2021_115_MOESM2_ESM.jpg]

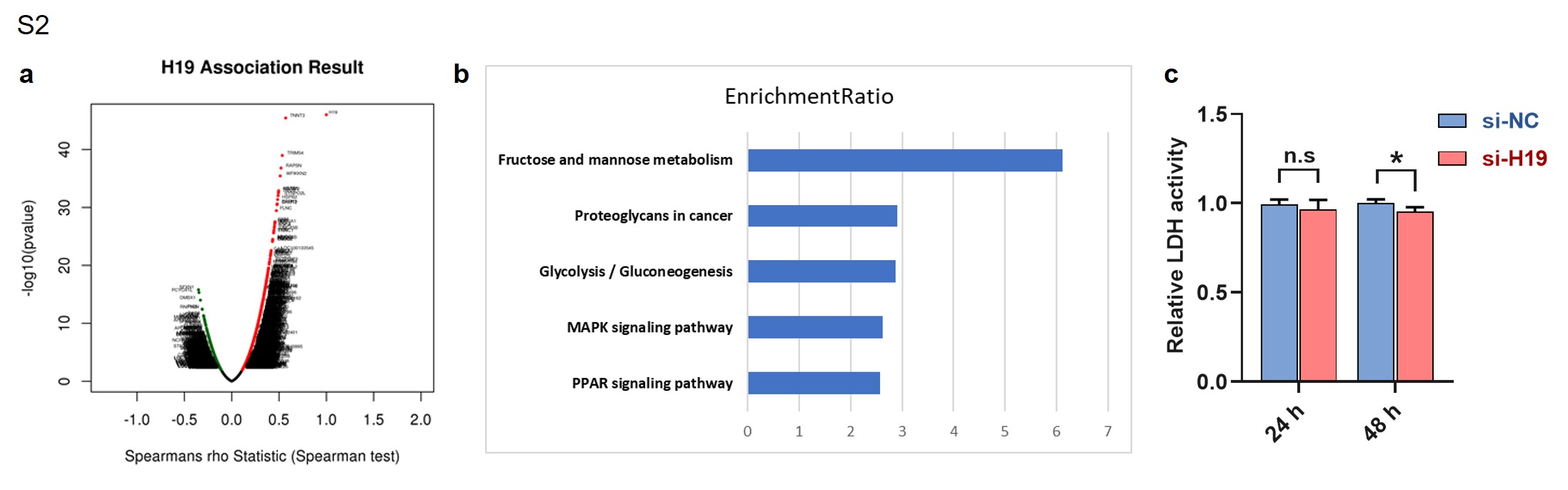

Supplement: Supplementary file 3 — Figure S2 [file 41368_2021_115_MOESM3_ESM.jpg]

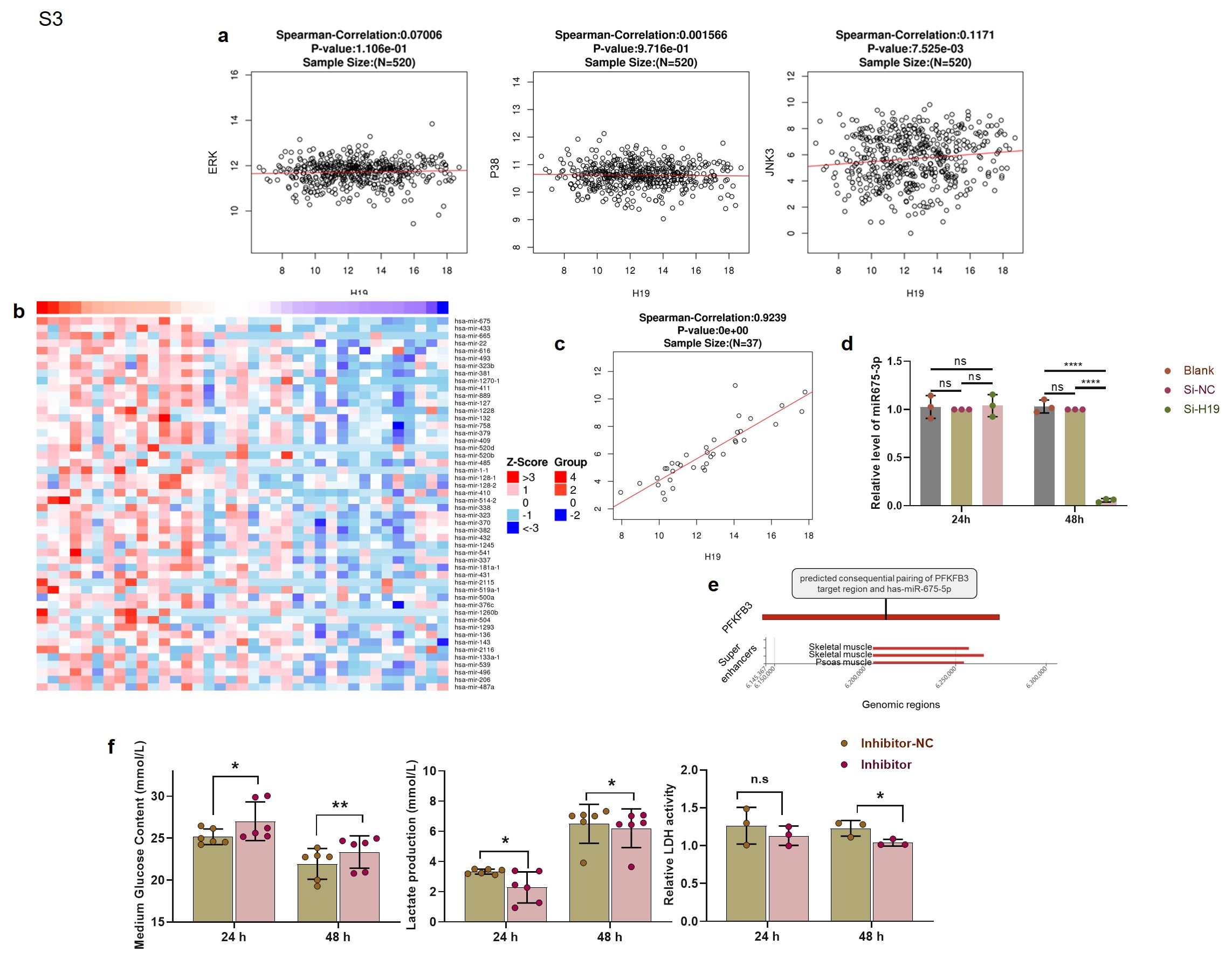

Supplement: Supplementary file 4 — Figure S3 [file 41368_2021_115_MOESM4_ESM.jpg]

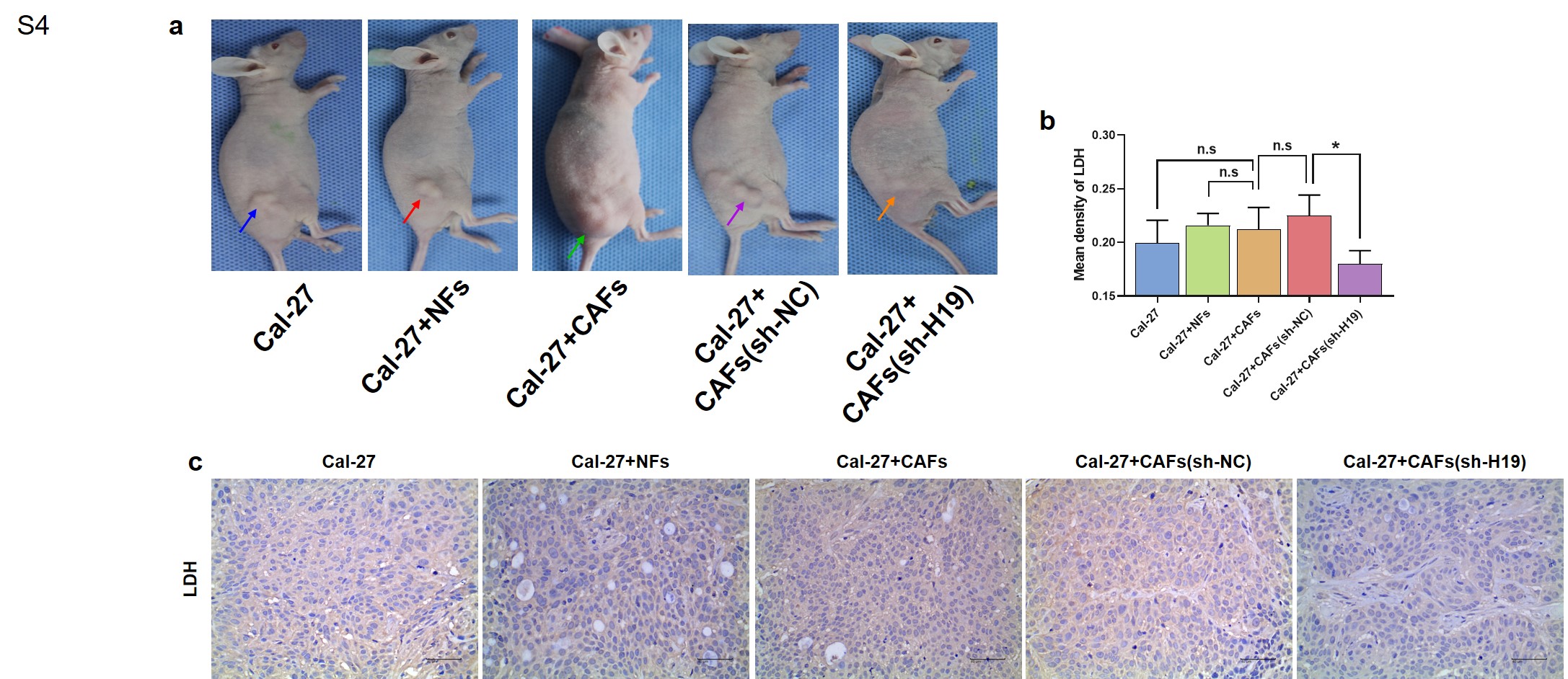

Supplement: Supplementary file 5 — Figure S4 [file 41368_2021_115_MOESM5_ESM.jpg]
